# *Cirsium setosum* Extract-Loaded Hybrid Nanostructured Scaffolds Incorporating a Temperature-Sensitive Polymer for Mechanically Assisted Wound Healing

**DOI:** 10.3390/pharmaceutics17050660

**Published:** 2025-05-17

**Authors:** Xiaojing Jiang, Shaoxuan Zhu, Jinying Song, Xingwei Li, Chengbo Li, Guige Hou, Zhongfei Gao

**Affiliations:** 1 Key Laboratory of Medical Antibacterial Materials of Shandong Province, School of Pharmacy, Binzhou Medical University, Yantai 264003, China; xiaojing199308@163.com (X.J.); skycitymeet@gmail.com (S.Z.); 13605453363@163.com (J.S.); xingweili2024@163.com (X.L.); lichengbo@bzmc.edu.cn (C.L.); 2 Key Laboratory for Agriculture Microbiology, Department of Microbiology, College of Life Science, Shandong Agricultural University, Tai’an 271018, China

**Keywords:** *Cirsium setosum*, temperature-response, biocompatibility, wound healing, hemostasis

## Abstract

**Background/Objectives**: *Cirsium setosum* (commonly known as thistle) is a traditional Chinese medicinal plant with significant therapeutic potential, exhibiting hemostatic, antioxidant, and wound-healing properties. Electrospinning offers a versatile platform for fabricating nanoscale scaffolds with tunable functionality, making them ideal for drug delivery and tissue engineering. **Methods**: In this study, a bioactive extract from thistle was obtained and incorporated into a thermosensitive triblock copolymer (PNNS) and polycaprolactone (PCL) to develop a multifunctional nanofibrous scaffold for enhanced wound healing. The prepared nanofibers were thoroughly characterized using Fourier-transform infrared spectroscopy (FTIR), contact angle measurements, thermogravimetric analysis (TGA), and tensile fracture testing to assess their physicochemical properties. **Results**: Notably, the inclusion of PNNS imparted temperature-responsive behavior to the scaffold, enabling controlled deformation in response to thermal stimuli—a feature that may facilitate wound contraction and improve scar remodeling. Specifically, the scaffold demonstrated rapid shrinkage at a physiological temperature (38 °C) within minutes while maintaining structural integrity at ambient conditions (20 °C). In vitro studies confirmed the thistle extract’s potent antioxidant activity, while in vivo experiments revealed their effective hemostatic performance in a liver bleeding model when delivered via the composite nanofibers. Thistle extract and skin temperature-responsive contraction reduced the inflammatory outbreak at the wound site and promoted collagen deposition, resulting in an ideal wound-healing rate of above 95% within 14 days. **Conclusions**: The integrated strategy that combines mechanical signals, natural extracts, and electrospinning nanotechnology offers a feasible design approach and significant technological advantages with enhanced therapeutic efficacy.

## 1. Introduction

The cutaneous barrier, recognized as the largest exposed organ in the human body, serves as a critical anatomical interface that maintains physiological homeostasis while protecting internal tissues from external insults, thereby preserving overall organismal integrity [1,2]. Despite its protective function, the skin remains susceptible to physical trauma, radiation, and chemical agents, which form wounds andinjuries. Commonly, the entire wound-healing process involves four intricately coordinated and sequential phases: hemostasis, inflammation, proliferation, and remodeling. However, even with the skin’s innate regenerative capacity, complications such as excessive bleeding, infection, and prolonged inflammation frequently occur, potentially leading to impaired healing or chronic wounds [3]. To address these challenges in the complex and dynamic wound microenvironment, nanofiber-based wound dressings have emerged as an increasingly sophisticated and continually evolving therapeutic strategy [4,5,6].

Electrospinning is commonly considered as a versatile approach for preparing integrated nanofibers with bioactive therapeutics [7,8,9]. Electrospun nanofibers exhibit exceptional structural and compositional similarities to the native skin extracellular matrix (ECM), featuring interconnected nano-networks with high surface-area-to-volume ratios [10]. The nanofibers also provide an ideal platform to delivery active molecules, such as antitumor medications, antibacterial, and antioxidant agents [11,12,13]. The growing emphasis on sustainable biomedicine has driven the incorporation of plant-derived bioactive extracts into nanofibrous systems, combining multifunctional therapeutic effects with inherent biocompatibility. Javier et al. [14] developed a nanostructured PCL electrospun mat with *Plantago major* L. extract, demonstrating significant antibacterial activity against methicillin-sensitive and resistant *S. aureus* and *E. coli* while promoting wound healing. Maleki et al. [15] engineered polyvinyl alcohol/chitosan nanofibers functionalized with *Zingiber officinale* and *Thymus vulgaris* extracts to accelerate infected wound repair. Nowadays, such rational and innovative utilization of natural products align with the principles of environmental sustainability [16], while offering cost-effective solutions for wound care management.

*Cirsium setosum*, a dual-purpose Chinese herb with both medicinal and edible applications, is rich in bioactive compounds including flavonoids, alkaloids, phenols, and sterols [17]. These phytochemicals demonstrate excellent hemostatic, antioxidant, antibacterial, anti-inflammatory, and hypoglycemic activities [18,19,20,21]. Some animals, as well as humans, have been observed applying crushed *Cirsium setosum* directly to injuries. Mechanistic studies reveal that these therapeutic effects are mediated through modulation of key signaling proteins, such as SRC, EGFR, AKT1, MAPK3, and GSK3B [22]. Recent advances have explored various pharmaceutical formulations of *Cirsium setosum*, such as hydrogels, carbon dots, and membranes, to expand its clinical applications. Geng and co-workers [23] developed a convergent hydrogel incorporating *Cirsium setosum* extracts for hemostasis and surgical anti-infections in the skin flap, wound defect, and femur fracture model. Luo and colleagues [20] discovered the water-soluble carbon dots from aqueous extracts of *Cirsium setosum*, which can stimulate the extrinsic blood coagulation system to exert an outstanding hemostatic effect. However, the incorporation of *Cirsium setosum* extracts into electrospun nanofibers for advanced wound therapy remains largely unexplored.

While nanofiber dressings relying solely on pharmacological activity demonstrate therapeutic potential, their clinical efficacy may be inherently limited. In recent years, it has become increasingly evident that the development of intelligent responsive materials offers expanded opportunities for the application of electrospun biomaterials in wound management. Temperature-sensitive polymers, particularly poly(*N*-isopropylacrylamide) (PNIPAM) exhibit unique temperature variations mediated by reversible hydration-dehydration transitions and hydrophobic interactions at the molecular level [24]. By leveraging this property, controlled mechanical traction forces can be applied to actively promote wound contraction.

**Figure 1:**
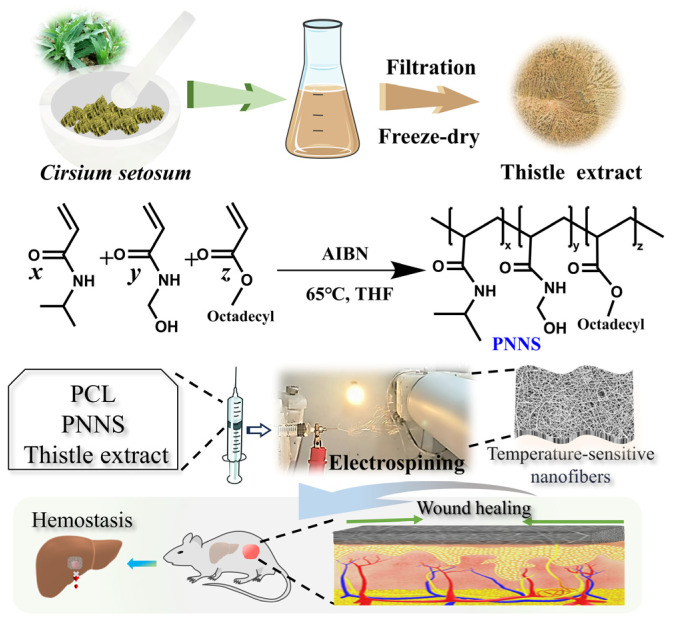
The process of thistle extract-loaded electrospun nanofibers incorporating a temperature-responsive PNNS for accelerating skin-wound healing.

In this work, water-soluble *Cirsium setosum* (thistle) extract was firstly prepared by a maceration method. The thistle extract and self-made temperature-sensitive polymerwere incorporated into the PCL matrix to fabricate electrospun nanostructured scaffolds (PNPT) for hemostasis and mechanically-assisted wound healing. A ternary solvent system of acetic/formic acid was employed to ensure the homogeneous dissolution of the hydrophilic extract, hydrophobic PCL, and thermosensitive PNNS, resulting in uniform fiber morphology with consistent diameter distribution. The PNPT scaffold demonstrated remarkable temperature-dependent behavior, exhibiting significant shape contraction at physiological temperature (38 °C) while maintaining structural integrity at ambient conditions (20 °C). This thermos-responsive characteristic enables the contraction of surrounding skin tissue around an open wound. The nanofibers possessed feasible mechanical tensile strength, thermal stability, and surface hydrophilicity. The results also illustrate its effective hemostatic effect and show an enhancement in its free radical scavenging capacity. Furthermore, demonstrating exceptional cytocompatibility and biocompatibility, the nanofiber mat promotes cutaneous wound healing by inhibiting inflammation, facilitating collagen deposition, and enhancing epidermal regeneration, thus serving as a promising therapeutic candidate and reference model for designing functional wound dressings with integrated properties (Figure 1).

## 2. Materials and Methods

### 2.1. Materials

Poly-ε-caprolactone (PCL, Mw: 80,000 g/mol) was purchased from Shenzhen Esun Industrial Co., Ltd. (Shenzhen, China). The triblock thermosensitive polymer, poly (*N*-isopropylacrylamideco-*N*-hydroxymethylacrylamide-co-octadecyl acrylate) (PNNS, M_w_ = 2176 Da) was prepared following our previous methods [25] and was characterized by a lower critical solution temperature (LCST) of 36.1 °C. Buffer Saline (PBS, pH 7.2–7.4) powder, trypsin, penicillin-streptomycin solution and a CCK-8 kit were provided by Servicebio Biotech, Co., Ltd. (Wuhan, China). Dulbecco’s Modified Eagle Media (DMEM) was purchased from Cytiva Co., Ltd. (Marlborough, MA, USA). 1,1-diphenyl-2-picrylhydrazyl (DPPH) and 2, 2′-azino-bis (3-ethylbenzothiazoline-6-sulfonic acid) (ABTS) were obtained from Shanghai Macklin Biochemical Technology Co., Ltd. (Shanghai, China). Other reagents were commercially available and were used directly without further treatment, unless otherwise specified.

### 2.2. Preparation of Cirsium Setosum Extract

*Cirsium setosum* was meticulously cleaned, dried, and ground into a fine powder to prepare the extract using the maceration method. Specifically, the thistle powder (30 g) was uniformly dispersed in a water–ethanol (volume ratio = 1:1, 2 L) mixed solvent at room temperature, stirring for 48 h. Subsequently, the extract solution was filtered, evaporated, and freeze-dried to obtain a yellowish-brown substance (3.28 g; yield: 10.93%).

### 2.3. Generation of Composite Nanofibers

Acetic/formic acid (weight ratio = 1:2) was selected as the spinning solvent based on Al-Kaabi et al.’s study [26]. More specifically, 3.5% (*w*/*v*) of PNNS and 7% (*w*/*v*) of PCL was dissolved into the above solvent to obtain the PNP electrospinning solution. A total of 3.0% (*w*/*v*) of the thistle extract was added into the PNP solution to prepare the PNPT electrospinning solution. Next, the resulting spinning solution was drawn into a 10 mL syringe equipped with a conductive needle (20 G, 0.6 mm) with a pump rate of 0.0017 mm/s. The distance between positive and negative electric fields was established at approximately 13–14 cm. Electrospinning was conducted at a temperature of 25 ± 2 °C and a relative humidity of 35 ± 2%. After applying a voltage at 14–15 kV for 8 h, the nanofibers were removed from the rolling receiver and then dried for 24 h.

### 2.4. Basic Characterization

#### 2.4.1. Scanning Electron Microscopy (SEM)

The surface of the nanofibers was electroplated with gold in an argon atmosphere. Subsequently, photographs of fiber morphology were collected using a scanning electron microscope (EVO LS15, Carl Zeiss, Oberkochen, Germany) at an accelerating voltage of 10 kV. The average fiber diameter was measured and calculated by randomly selecting fifty fibers from the SEM images utilizing the Image J software (version 1.46, NIH, Bethesda, MD, USA).

#### 2.4.2. Fourier Transform Infrared (FTIR)

Electrospun mats (PNP, PNPT) were analyzed using an infrared spectrometer (Nicolet iS50, Thermo Fisher Scientific, Waltham, MA, USA) to determine their chemical composition. The spectrum was scanned at a resolution of 4 cm^−1^ across a range of 4000–500 cm^−1^ for 32 scans.

#### 2.4.3. Static Contact Angle Measurement

Surface hydrophobicity or hydrophilicity was assessed by measuring the contact angles between the liquid/gas and the materials’ surface. Deionized water, a commonly used surface liquid, was used for the contact angle measurement (2.0 μL each time). The electrospun nanofibers (2.0 cm × 2.0 cm) were positioned within the field of view (FOV) of the camera in the contact angle testing instrument (SZ-CAMD33, Shanghai Sunzern, Shanghai, China). Photographs were captured from the moment droplets first made contact with the materials’ surface until complete penetration occurred.

#### 2.4.4. Thermo-Responsive Assay

As outlined in previous methods [25], nanofiber sheets measuring about 2 cm × 2 cm were precisely cut and transferred into 20 mL of distilled water. The samples were then subjected to treatments at temperatures of 20 °C, 38 °C, and 40 °C for a duration of 10 min each.

#### 2.4.5. Thermal Behavior (DSC and TGA)

The thermal properties of nanofibers were determined by a synchronous thermogravimetry (TGA) and differential scanning calorimetry (DSC) analyzer (TGA/DSC3+, Mettler Toledo, Greifensee, Switzerland) ranging from 25.0 °C to 800.0 °C in an ultra-pure nitrogen atmosphere with a heating rate set to 10.0 °C/min.

#### 2.4.6. Fracture Tensile Testing

Samples of a known thickness were punched into a dumbbell configuration (20 mm × 2 mm) and subjected to vertical loading using a microcomputer-controlled electronic universal mechanical instrument (WDW-500DS, Jinan Hensgrand Instrument, Ji’nan, China) at 2 mm/min. The effective stretching distance was established at 20 mm. The testing procedure was performed at room temperature with a humidity of 40–50%.

### 2.5. Antioxidant Assay

The antioxidant assay was conducted in accordance with the established protocol [27] with minor modifications. Briefly, 5 mL of 0.1 M DPPH ethanol solution was mixed with 50 mg of PNP or PNPT nanofibers and incubated away from light for 30 min using DPPH solution alone as the negative control. For the ABTS assay, the cationic free radical was prepared by the oxidation of ABTS (0.0374 g) with potassium sulfite (0.0066 g) into deionized water (30 mL), followed by 10 h of dark incubation at room temperature. The following determination was consistent with the DPPH method. The optical density (OD) values were measured at wavelengths of 517 nm for DPPH and 734 nm for ABTS, respectively. When free radical solutions were incubated with the sample, the lightening of the solution color indicated effective free radical scavenging, which was correlated with a decrease in the OD value. The decrease in OD values indicates that the material exhibits better antioxidant properties. Each experiment was repeated three times. The elimination of the free radical was calculated as the formula: Elimination (%) = (*OD_control_* − *OD_sampl_*_e_)/*OD_contro_*_l_ × 100%, where *OD_sampl_*_e_ and *OD_control_* represent the OD values of the experimental groups and control groups, respectively.

### 2.6. Hemostatic Assay In Vivo

The hemostatic efficacy of the nanofiber dressings was evaluated using a standardized murine liver hemorrhage model. Following anesthesia induction, the mouse underwent an abdominal incision, which exposed its liver. A controlled hemorrhage was induced by creating a uniform puncture wound (0.8 mm diameter) in the left lateral lobe using a sterile 26-gauge needle. Immediately, a pre-weighted sample was covered on the wound sites to stop the bleeding. The hemorrhage volumes and macroscopic photos were recorded during the hemostatic process. The experiments were repeated three times for each group.

### 2.7. Cell Viability and Hemolytic Assessment

The L929 fibroblasts cell line was selected to assess the cytocompatibility of the nanofiber dressings. The nanofibers (6 cm^2^) were completely immersed in a DMEM serum-free medium (1 mL) for 24 h at 37 °C to obtain the leaching solution following sterilization with a 0.22 μm filter. When co-cultured with the leaching solution at various dilution levels for 24 h, cell survival was evaluated using the CCK-8 kit. Each group underwent three parallel repetitions. Cell viability was determined by calculating the ratio of OD values between the experimental and control groups.

The hemolysis rate was conducted to evaluate the hemocompatibility in direct contact with blood cells. Fresh whole blood from mice was centrifuged at 1000 rpm for 5 min to separate the red blood cells, which were then washed with sterile saline until the supernatant became clear. A mixture of nanofibers (50 mg) and diluted blood cells (1 mL) was incubated at 37 °C for 1 h, followed by centrifugation. A total of 100 µL of the supernatant was transferred to a 96-well plate to determine the OD values at 540 nm using a microplate reader. Distilled water and sterile saline served as positive and negative controls, respectively. The experiments were repeated three times. The hemolysis rate (%) = *(OD_s_* − *OD_n_*)/(*OD_p_* − *OD_n_*) × 100%, where *OD_s_*, *OD_p_*, and *OD_n_* are the OD values of nanofibers, positive, and negative groups, respectively.

### 2.8. Healing Effects on the Full Defect Wound Model

BALB/c mice (8 weeks, 19–22 g) were obtained from Shandong Pengyue Experimental Animal Technology Co., Ltd., (Jinan, China). All procedures were performed with the approval of the Committee on the Ethics of Animal Experiments of Binzhou Medical University (protocol number: 2021–315) and the National Institutes of Health Guide for the Care and Use of Laboratory Animals. The animals were divided into four groups based on their dressings treatment: Gauze, Tegaderm (a commercial breathable film from 3M company, Saint Paul, MN, USA), PNP, and PNPT groups. Each group consisted of three mice at every time point. Specifically, a full defect wound (approximately 70 mm in diameter) was created on the mouse’s back skin using surgical scissors after sufficient hair removal and disinfection. Subsequently, each defect site was covered with one of the aforementioned dressings and secured with an elastic bandage and medical tape. Treated mice were housed individually in separate cages, and the dressings were changed every two days. Photos of the treated wound were captured with a digital camera on postoperative days (POD) 0, 3, 7, 10, and 14. The wound area for each group was measured with ImageJ software (version 1.46, NIH, Bethesda, MD, USA) and calculated as the formula Healing rate (%) = (*A*_0_ − *A_n_*)/*A*_0_ × 100%, where *A*_0_ and *A_n_* are the measured original area and treated wound area at the aforementioned scheduled time points, respectively.

To evaluate the inflammation response, collagen deposition and other histopathological characteristics, harvested wound tissues were fixed in 4% paraformaldehyde and subsequently sectioned into paraffin slides for hematoxylin and eosin (HE) staining, as well as for Masson’s trichrome staining.

### 2.9. Statistical Analysis

All original data were processed using a one-way ANOVA based on the mean values and standard deviations (SD) in the OriginPro software (version 10.2.0, OriginLab Corporation, Northampton, MA, USA). Statistically significant levels were considered as *p* * < 0.05, *p* ** < 0.01, *p* *** < 0.001.

## 3. Results and Discussion

### 3.1. Preparation and Characterization of Electrospun Nanofibers

#### 3.1.1. Fiber Morphology

**Figure 2:**
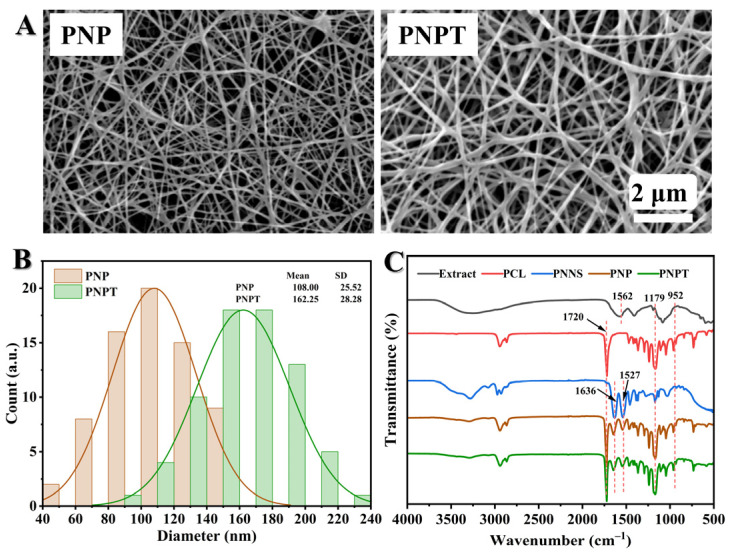
Structural and morphology characterization of PNP and PNPT nanofibers. (**A**) Representative SEM images of PNP (**left**) and PNPT (**right**) nanofibers. (**B**) The fiber diameter distribution of PNP and PNPT. (**C**) FTIR spectra of the thistle extract, PCL, PNNS, PNP, and PNPT.

The electrospun nanofibers were designed to structurally and functionally mimic the native ECM, thereby promoting cell proliferation and tissue regeneration. The morphology of nanofiber scaffolding was influenced by optimizing key electrospinning parameters including applied voltage, distance, infusion rate, and the concentration of the spinning solution. Finally bead-free continuous fibers were prepared according to the optimal machine parameters (voltage, 14 kV; distance, 13 cm; pump speed, 0.0017 mm/s with a 10 mL standard syringe). While the PNP fibers displayed smooth, uniform topography with occasional micrometer-scale cracks, the PNPT fibers (incorporating thistle extract) exhibited significantly increased surface roughness and porous network formation (Figure 2A). This morphological alteration suggests that the hydrophilic extract modulates the polymer’s self-assembly behavior during electrospinning. The ternary solvent system (acetic/formic acid, 1:2 m/m) effectively dissolved both the hydrophilic extract and hydrophobic PCL/PNNS components, enabling homogeneous fiber formation. Quantitative analysis demonstrated that PNPT fibers (162.25 ± 25.52 nm) had larger diameters than PNP fibers (Figure 2B), attributable to the incorporated extract. Both fiber types (50–300 nm diameter range) closely approximated the dimensional characteristics of native dermal collagen fibrils [28].

#### 3.1.2. FTIR Spectra Analysis

The chemical structure was analyzed using FTIR to investigate the functional groups present in the prepared nanofibers. As shown in Figure 2C, the spectrum of the thistle extract showed a broad peak at 3400–3000 cm^−1^ and 1179–952 cm^−1^, which can be attributed to hydroxyl group (O-H) stretching and C-O-C stretching, respectively, and possibly to phytochemicals (e.g., flavonoids or polyphenols). The bending vibration of O-H resulted in a signal at 1562 cm^−1^ observed in the extract, PNNS, PNP, and PNPT groups. For PCL, a peak was noted at 1720 cm^−1^, corresponding to the C=O stretching vibration. Additionally, in the PNNS, PNP, and PNPT groups, characteristic peaks for amide I and amide II were detected at 1636 cm^−1^ and 1527 cm^−1^, respectively [25]. Specifically analyzing the PNPT curve revealed peaks associated with PCL, indicating that PCL was incorporated into the PNPT fibrous membrane through physical mixing, according to the existing literature [26]. The presence of peaks at 1562 cm^−1^, 1179 cm^−1^, and 952 cm^−1^ from the extract within the PNPT suggests the thistle was generated into the PNPT composite nanofibers.

#### 3.1.3. Contact Angle Analysis

**Figure 3:**
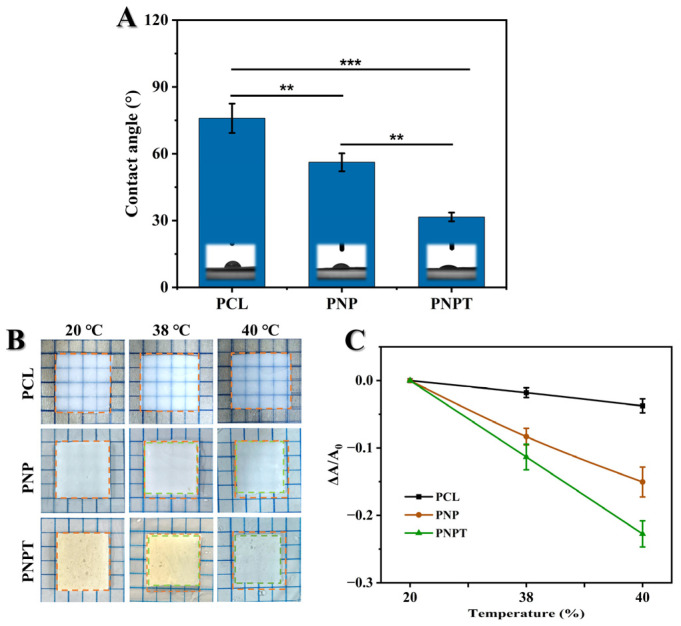
(**A**) Static water contact angles for the electrospun nanofibers. (**B**) Representative images of temperature-responsive shrinkage of nanofibers immersed in 20, 38, and 40 °C water. Wireframe in yellow and green represents the shape of original and heated nanofiber, respectively. (**C**) The relative change in surface area caused by temperature variations. *p* ** < 0.01, *p* *** < 0.001, n = 3.

The surface wettability of nanofibers is crucial for the absorption and exudation, drug release kinetics, and the efficacy of therapeutic tissue repair during the wound-healing process. Surface hydrophobicity or hydrophilicity was characterized through water contact angle (WCA) measurements. As depicted in Figure 3A, PCL exhibited the highest level of surface hydrophobicity. The addition of PNNS resulted in an increase in the contact angle of PNP to 56° compared to that of PCL (76°), which can be attributed to the rapid hydration properties of PNNS, thereby inducing a wetting effect. PNPT exhibited a significant decrease in WCA (32°) upon incorporating thistle extract, which promotes a favorable wound environment and enhances wound repair through the release of thistle components. The temperature-induced disruption of hydrogen bonds and the aggregation of PNNS hydrophobic chains, along with the extract released, led to a more pronounced contraction of PNPT compared to PNP. Therefore, we hypothesize that the extract within the nanofibers may possess burst release properties at a normal physiological temperature (37 °C), which is advantageous for acute wounds recovery.

#### 3.1.4. Thermo-Responsive Properties

The triblock thermosensitive polymer PNNS was incorporated into the nanofibers to enhance temperature-responsive mechanical contraction, thereby facilitating the centripetal closure of wounds [29]. Here, we designed a straightforward experiment to simulate in vitro the contraction behavior of the fiber membrane in response to temperature variations. The relative contracting area of PCL fiber had no significant changes when heated from 20 °C to 40 °C. Upon exposure to increasing temperature, both PNP and PNPT fibers exhibited varying degrees of active contraction (Figure 3B). This phenomenon can be attributed to the hydrophobic interactions between the isopropyl and long-chain alkyl groups present on the PNNS, which led to the aggregation of molecular chains and phase transition [30]. Notably, thistleextract-doped fibers, PNPT, exhibited more pronounced area shrinkage both at 38 °C and 40 °C compared to PNP (Figure 3C). This effect may be due to the detachment of the extract within fibers, resulting in weakened hydration-related hydrogen bonding while relatively strengthening the hydrophobic interactions. These results indicate that PNPT nanofibers exhibit excellent temperature-responsive contraction performance.

#### 3.1.5. Thermogravimetric Performances

**Figure 4:**
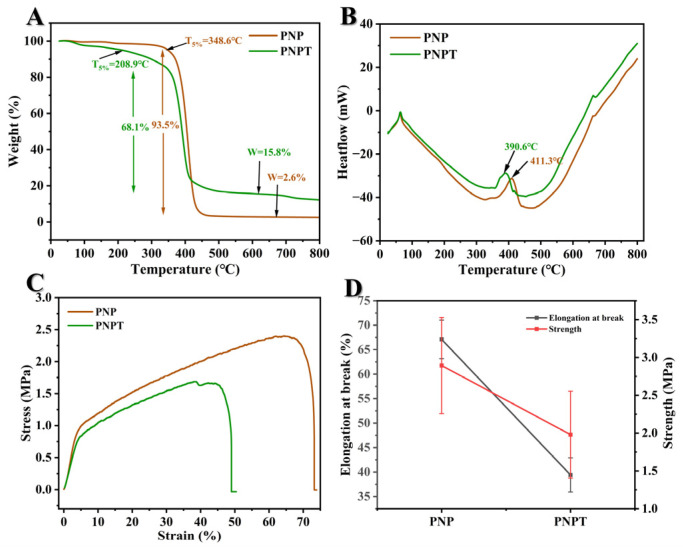
Thermogravimetry and mechanical tensile evaluation of nanofibers. TGA (**A**) and DSC (**B**) curves of PNP and PNPT fibers. (**C**) Stress versus strain curves. (**D**) Elongation at break and maximum strength based on three repeated experiments, n = 3.

TGA and DSC analyses were conducted to evaluate the crystalline changes, melting behavior, and component decomposition under programmed temperature conditions [31]. The TGA curves for PNP and PNPT nanofibers are presented in Figure 4A, revealing distinct behaviors characterized by the initial weight loss temperature (T_5%_) and the heating residue. PNPT possessed a T_5%_ at 208.9 °C while PNP showed a significantly higher value of 348.6 °C due to the separation of the hydrate within the thistle extract. At the final stage of the programmed temperature, PNPT still exhibited a significantly higher residue weight of approximately 15.8%, in contrast to PNP’s residue weight of only 2.6%. Concurrently, as shown in Figure 4B, the DSC results indicated a prominent peak at 390.6 °C for the PNPT group, which is lower than that observed in the PNP group (411.3 °C). This suggests thistle extract contains components that are susceptible to thermal decomposition, while also exhibiting high stability.

#### 3.1.6. Mechanical Strength Properties

Suitable strength properties can provide a favorable biomechanical microenvironment and ensure the accessibility of practical applications. A study reported that the tensile strength of normal skin is typically around 2.5 MPa, with an elongation at the break of approximately 70% [32]. As illustrated in Figure 4C,D, thistle extract-loaded nanofibers showed weaker mechanical tensile property but a similar modulus compared to nanofibers without extract. More specifically, the strength and elongation at the break of PNPT were measured at 1.98 ± 0.57 MPa and 39.41 ± 3.50%, respectively, while PNP demonstrated values of 2.89 ± 0.64 MPa and 67.09 ± 3.95%. The differences in physical interaction (hydrogen and hydrophobic bonding) between PCL/PNNS and the extract may significantly contribute to reducing the cross-section density of each nanofiber. This membrane may be suitable for low-tension wounds (such as facial or abdominal wounds) but not for high-mobility areas (like joints or palms). Although PNPT is weaker than skin, excessive stiffness (>5 MPa) could impede natural tissue movement and delay healing. PNPT falls within an acceptable yet improvable range of 1–5 MPa (similar to commercial silicone gels and polyurethane films). To enhance the mechanical performance of PNPT without compromising biocompatibility, strategies such as nanocomposite reinforcement, crosslinking technology, hybrid material, and advanced mechanical structural optimization could be employed to achieve a balance among strength, elasticity, and bioactivity. Therefore, mechanical strength would be a critical consideration for further optimizing parameters and rationally designing nanofiber dressings.

### 3.2. Antioxidant Capacities and Hemostatic Efficiency

**Figure 5:**
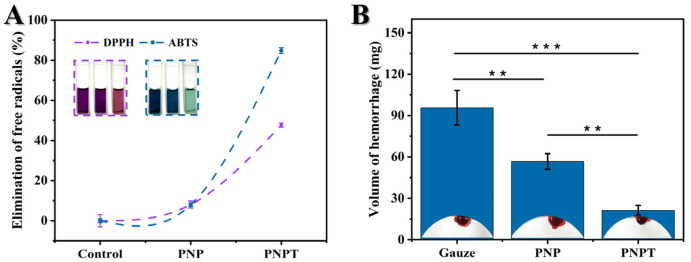
Antioxidant and hemostatic capacities of prepared nanofiber dressings. (**A**) Clearance levels of neutral (DPPH) and cationic free radicals (ABTS) in various groups (n = 3). (**B**) The PNPT effectively reducing liver hemorrhage (n = 3, *p* ** < 0.01, *p* *** < 0.001).

*Cirsium setosum*, known for its antioxidant activities, can be beneficial for treating inflammatory and chronic disease [21,33], which prompts us to investigate the free radical scavenging capacity of the thistle-extract-doped nanofibers in this study. DPPH and ABTS are commonly used as the carriers for neutral and cationic free radicals, respectively, to simulate antioxidant experiments in vitro. The results are shown in Figure 5A. PNPT showed statistical significance compared to both the control and PNP fibers, effectively eliminating about 47% of the DPPH free radicals. For the cationic free radical from ABTS, elimination exceeded 80% in the PNPT group, displaying that the thistle extract utilized in this work retains a significant antioxidant capacity within the nanofibers.

Similarly, the hemostatic efficiency of *Cirsium setosum* has been found to be remarkable among numerous traditional Chinese herbs [34,35]. A live hemorrhage model in vivo was employed to study the hemostatic efficiency of thistle extract-loaded nanofibers. Treatment with PNPT nanofibers resulted in a significant reduction in blood volume compared to both the control and PNP group (*p* < 0.001, shown in Figure 5B). Additionally, the PNP nanofibers displayed less hemorrhage than the control group (*p* < 0.01) due to hydration from the presence of PNNS. The antioxidant capacities and hemostatic efficiency demonstrated by the above results provide foundational therapeutic effects on curing cutaneous defects.

### 3.3. Cytocompatibility and Hemocompatibility

**Figure 6:**
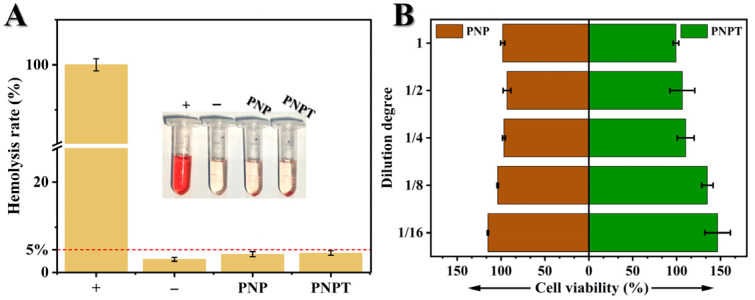
Biocompatibility of PNP and PNPT nanofibers. (**A**) Hemolysis rate of water (+), sterile saline (−), PNP and PNPT nanofibers (n = 3); insert: photograph of the sedimented blood cells after centrifugation. (**B**) Cell viability of leaching solution of PNP and PNPT nanofibers with different dilutions after culturing with L929 fibroblasts (n = 3).

Good biocompatibility is an essential prerequisite for ensuring the safety of in vivo treatments [36]. As shown in Figure 6A, PCLand PNNS-generated nanofiber PNP exhibited a hemolysis rate of 4.04 ± 0.56%, revealing a promising hemocompatibility upon the incorporation of PNNS into PCL nanofibers. Furthermore, PNPT complies with the standard requirements outlined in ISO 10993–4 with a hemolysis rate of less than 5% [37,38]. Additionally, L929 fibroblast line was cultured with leaching liquid to evaluate cell viability (Figure 6B). It is worth noting that serum contains a variety of components, including growth factors, hormones, and proteins, which may obscure the direct effects of material extracts, potentially altering their concentration, activity, or stability. In biocompatibility assays, serum may neutralize toxic substances released by the material, masking the actual effects. By contrast, a serum-free medium allows for a more direct assessment of how material extracts influence cell survival, proliferation, or function. After 24 h of incubation, no significant differences were observed between PNP and PNPT across various leaching dilutions, demonstrating the non-toxic effects of the prepared nanofibers and confirming the suitability of the thistle extract for use as wound dressings.

### 3.4. Wound Healing Promoting Efficacy In Vivo

**Figure 7:**
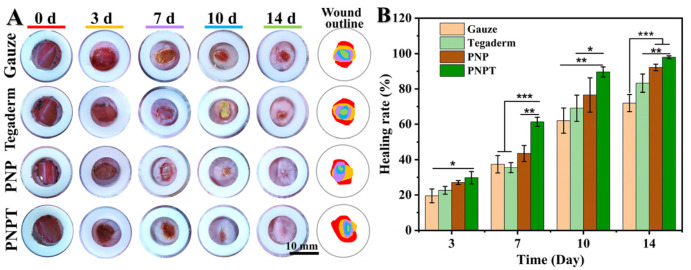
Macroscopic observation of the wound-healing process. (**A**) Photos of healing wound tissue and merging wound outline mapping on POD 0, 3, 7, 10, and 14. (**B**) The healing rate compared to the original wound on the postoperative days. *p* * < 0.05, *p* ** < 0.01, *p* *** < 0.001, n = 3.

The characterization, activity validation, and biocompatibility assessment support the utilization of thistle-extract-loaded electrospun nanofibrous scaffolds for mechanically-assisted wound healing in a full-thickness skin defect model. Figure 7A displays the wound closure with various treatments (Gauze, Tegaderm, PNP, and PNPT) on POD 0, 3, 7, 10, and 14. Overall, PNPT nanofibers were observed to promote scab formation during the early stage of wound healing, accelerate the healing process within 7 days, and gradually facilitate epidermal remodeling after this period. On POD 3, PNP and PNPT showed healing rates of 27.05 ± 1.11%, 29.78 ± 3.49%, respectively, compared to the Gauze (19.49 ± 3.91%) and Tegaderm groups (22.62 ± 2.20%) (shown in Figure 7B). This disparity may be attributed to the synergistic effects of temperature-sensitive shrinkage associated with PNNS combined with the active properties of the extract in the inflammatory phase of wound healing. More specifically, with regard to POD 14, there was a significant difference observed between the PNPT group when compared to the PNP (*p* < 0.01), Gauze (*p* < 0.001), and Tegaderm (*p* < 0.001) groups. Additionally, it was observed that PNP without thistle extract exhibited enhancing healing effects relative to the Gauze and Tegaderm groups, but it was not as remarkable as the PNPT group. Ultimately, a wound treated solely with PNPT progressed towards complete healing while other groups experienced challenges in achieving similar outcomes due to visible erythema and scarring issues present at that time point. These results collectively demonstrate excellent therapeutic efficacy in promoting wound healing.

**Figure 8:**
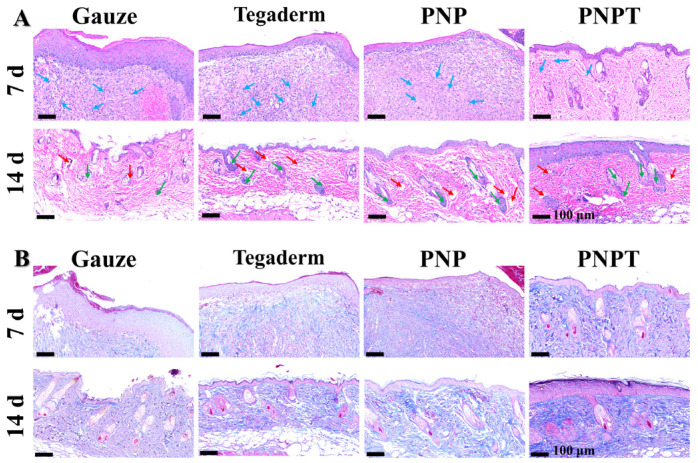
Histopathology assessment utilizing HE staining (**A**) and Masson’s trichrome staining (**B**). Blue arrows: inflammatory cells; red arrows: blood vessel; green arrows: newly formed hair follicles.

To evaluate the wound-healing effects of the nanofiber dressings, HE and Masson staining were conducted to observe the micromorphological details, with the results presented in Figure 8. On POD 7, wound tissue treated with PNPT exhibited less inflammation and more vascularization compared to the Gauze, Tegaderm, and PNP groups (Figure 8A). This finding suggests that thistle extract played a key role in inhibiting inflammation and promoting healing. With continuous treatment until day 14, all groups showed varying degrees of regeneration in blood vessels, epidermis, dermis, and skin appendages. The recovery tissues treated with PNP and PNPT appeared relatively organized compared to the other two groups, which resulted from the thermal response shrinkage performance of nanofiber membranes. Furthermore, Masson staining was employed to analyze the collagen deposition and distribution throughout the wound-healing process. On both days 7 and 14, the PNPT group exhibited significantly greater collagen deposition than the other groups. This may be due to the extract having reduced the inflammatory response while accelerating the transition from the inflammatory phase to the proliferative phase. Thistle extract possessed remarkable antioxidant and hydrophilicity, which contribute significantly to reducing inflammation and preventing scar hyperplasia.

Electrospun nanofibers in this study exhibit significant differences in material structure and functional characteristics when compared to the previously reported hydrogels [23]. We propose a design strategy involving temperature-responsive mechanical modulation and active natural extracts, validating the synergistic effects of the natural/synthetic hybrid system on wound healing. Specifically, the thermal contraction-induced physical stimulation and bioactive components of these systems play a critical role in facilitating the healing of chronic and open wounds. The hydrogels described in the existing literature are predominantly composed of synthetic polymers and antibacterial agents, characterized by their broad-spectrum anti-infection properties, adhesion capabilities, and moist wound-healing attributes. Their effectiveness largely depends on the antibacterial activity of chemical substances and their physical barrier function. In contrast, this study highlights unique features related to the development of intelligent response mechanisms and the incorporation of natural components, thereby establishing a complementary relationship with anti-infection hydrogels. Future research could explore combining these advantages to develop multifunctional dressings capable of providing mechanical stimulation and antibacterial activity and enhancing wound healing.

## 4. Conclusions

In this study, we developed a hybrid nanofibers network that incorporates a natural extract as an active component, and a biocompatible thermosensitive polymer, PNNS, as a mechanically-assisted healing material for enhanced wound management. The *Cirsium setosum* extract was meticulously prepared using an optimized maceration method with 50% ethanol aqueous solution, followed by rotary evaporation and freeze-drying to preserve its bioactive constituents. Thermosensitive PNNS (LCST= 36.1 °C) was synthesized through a radical copolymerization reaction. The *Cirsium setosum* extract-loaded nanofibers (PNPT) maintained structural integrity without bead formation or morphological defects, exhibiting a narrow diameter of 162.25 ± 25.52 nm to ensure consistent mechanical properties and thermal adaptability. The PNPT nanofibers demonstrated diverse shrinkage characteristics in response to varying temperatures, enabling the material to adapt to wound temperature fluctuations and potentially providing mechanical stimulation that promotes healing in open wounds. Furthermore, the extract enhanced the surface hydrophilicity of the nanofibers. Additionally, PNPT nanofibers possess remarkable antioxidant and hemostasis effects, along with expectant cellular compatibility and hemocompatibility. The hybrid nanofiber scaffolds facilitate the repair of cutaneous defects through inflammatory inhibition, re-epithelialization, and collagen deposition. In summary, the combination of *Cirsium setosum* extract with temperature-sensitive polymer presents a viable strategy for skin wound repair. Finally, establishing a robust system for screening the optimal bioactivity of natural extracts, as well as ensuring precise and effective drug release and periodic treatment when integrated into intelligent responsive materials, represents a promising research direction.

## Data Availability

The data that support the findings of this study are available from the corresponding authors upon reasonable request.
